# Auditory Mismatch Negativity in Youth Affected by Autism Spectrum Disorder With and Without Attenuated Psychosis Syndrome

**DOI:** 10.3389/fpsyt.2020.555340

**Published:** 2020-11-24

**Authors:** Giorgio Di Lorenzo, Assia Riccioni, Michele Ribolsi, Martina Siracusano, Paolo Curatolo, Luigi Mazzone

**Affiliations:** ^1^Laboratory of Psychophysiology and Cognitive Neuroscience, Department of Systems Medicine, University of Rome Tor Vergata, Rome, Italy; ^2^IRCCS Fondazione Santa Lucia, Rome, Italy; ^3^Child Neurology and Psychiatry Unit, Department of Systems Medicine, University of Rome Tor Vergata, Rome, Italy; ^4^Psychiatry Unit, Campus Bio-Medico University of Rome, Rome, Italy; ^5^Department of Biomedicine and Prevention, University of Rome Tor Vergata, Rome, Italy; ^6^Department of Biotechnological and Applied Clinical Sciences, University of L'Aquila, L'Aquila, Italy

**Keywords:** autism spectrum disorder, attenuated psychosis syndrome, mismatch negativity, EEG, pediatric

## Abstract

The present study investigates the differences in auditory mismatch negativity (MMN) parameters given in a sample of young subjects with autism spectrum disorder (ASD, *n* = 37) with or without co-occurrent attenuated psychosis syndrome (APS). Our results show that ASD individuals present an MMN decreased amplitude and prolonged latency, without being influenced by concurrent APS. Additionally, when correlating the MMN indexes to clinical features, in the ASD + APS group, we found a negative correlation between the severity of autistic symptoms and the MMN latency in both frequency (f-MMN *r* = −0.810; *p* < 0.0001) and duration (d-MMN *r* = −0.650; *p* = 0.006) deviants. Thus, our results may provide a more informative characterization of the ASD sub-phenotype when associated with APS, highlighting the need for further longitudinal investigations.

## Introduction

Autism spectrum disorder (ASD) is a neurodevelopmental condition characterized by an early onset of social communication deficit associated with a restricted and repetitive pattern of behaviors (1). Since socio-communication impairment is known as a core symptom of ASD, several studies are investigating the role of language difficulties on the central auditory process that, in turn, may be linked to an atypical processing of auditory stimuli, an auditory working memory deficit, and a disrupted sensory acoustic discrimination (2–5).

To better understand the auditory processing in neurodevelopmental disorders, mismatch negativity (MMN) has been widely investigated by basic researches on neurocognitive processes and central auditory and attentional mechanisms (4, 6–9). Specifically, MMN is a negative wave deflection localized in the fronto-central region (10), resulting from the brain's response to infrequent auditory stimuli (deviants) in repetitive stimuli (standards) (6, 11). MMN originates in the auditory and frontal cortex by the central auditory system based on neuronal auditory memory (12) and reflects pre-attentive processes, involving executive functions such as set-shifting ability and working memory (13–15). Thus, researchers have proposed MMN deficit as a marker of auditory processing impairment and consequent disruption in higher cognitive domains (e.g., attentional control and shifting, working memory) (7). Even in the ASD population, MMN has been used to identify auditory processing deficits, with conflicting results (16–18): a recent meta-analysis (17) on the topic shows that most of the available data report an MMN deficit in ASD individuals, suggesting an altered central ability in auditory discrimination within this population; specifically, Chen et al. (17) report shorter MMN latencies in autistic individuals compared to increased MMN latencies in subjects with Asperger syndrome (AS).

Abnormally decreased MMN has also been reported in other psychiatric disorders such as schizophrenia (19–24), especially in the early stages of the illness (7, 25–27). MMN has been widely investigated as a promising biomarker of conversion to psychosis (7, 26) and of remission (28) in individuals at a clinical high risk (CHR) and with attenuated psychosis syndrome (APS). To date, the majority of the available data reports a reduced amplitude of MMN in the at-risk adult population (29). In this context, Bodatsch et al. (30), evaluating the MMN paradigm in a young adult sample of 62 CHR subjects compared to 33 individuals with first-episode schizophrenia and 67 healthy controls (HC), concluded that MMN amplitude was significantly reduced in at-risk subjects who later converted to first-episode psychosis compared to non-converter individuals and to HC.

Despite distinct clinical and electrophysiological features, several studies have focused on the overlap between autistic symptoms and psychotic experiences in both adult and adolescent population (31–33). Longitudinal studies report that 20–50% of individuals with childhood-onset schizophrenia met the criteria for premorbid ASD (34–37). Moreover, a recent meta-analysis confirms that people affected with schizophrenia spectrum disorders (SSDs) show higher autistic symptoms compared to healthy controls (38). Similarly, in ASD individuals, the comorbid rates of SSDs ranging from 0 to 34.8% are reported, with higher rates of transition to SSDs in specific clinical subgroups such as AS and pervasive developmental disorder—not otherwise specified (PDD-NOS), categorized based on the Diagnostic and Statistical Manual of Mental Disorders—Fourth Edition (DSM-IV) (32); this evidence could suggest a possible increased vulnerability in the autistic population (32).

Moreover, it is well-known that individuals with ASD and SSDs share some clinical features, such as social difficulties, language impairment, and common cognitive features (e.g., weak central coherence deficit and set-shifting difficulties) (33, 39, 40).

Thus, in the recent years, research aiming to detect possible cognitive and neurophysiological linkages between these disorders has grown considerably (33, 41–43).

However, despite the evidence of this strong association between ASD and SSDs, to our knowledge, no previous study using a clinical and neurophysiological approach has yet evaluated the co-occurrence of both conditions within a pediatric sample.

The present study aimed to characterize, from a clinical and neurophysiological point of view, a sample of ASD participants (age range: 9–18 years) with attenuated psychosis syndrome (ASD + APS) in comparison with ASD patients without APS (ASD) and with healthy control (HC) groups, pointing to better defining the ASD phenotype when associated with other psychiatric conditions such as APS.

## Materials and Methods

### Participants

A total of 40 ASD individuals (age range: 9–18 years; age median: 13.67; eight females and 32 males), according to Diagnostic and Statistical Manual of Mental Disorders—Fifth Edition (DSM-5) criteria (1), were recruited from the Children Psychiatry Unit of the University Hospital Tor Vergata of Rome between January 2018 and July 2019.

A healthy control (HC) group of 20 individuals (age range: 9–18 years; age median: 13.04; eight females and 12 males), voluntarily recruited from a sport club, was also included.

The present study was approved by the Ethical Committee of our University Hospital, Fondazione Policlinico Tor Vergata (Register number 126/18), and informed consent was obtained from all legal holders of custody of both ASD and HC groups.

The adopted exclusion criteria were the presence of syndromic autism, intellectual quotient (IQ) equal or below 70, non-fluent speech, epilepsy, and other concurrent psychiatric or neurodevelopmental conditions (e.g., obsessive–compulsive disorder, attention deficit and hyperactivity disorder).

All participants underwent a clinical evaluation and electroencephalogram (EEG) to evaluate the MMN paradigm.

From the initial sample of 40 ASD individuals, three did not complete the clinical assessment and dropped out of the study. The final sample of 57 participants (37 ASD + 20 HC) was divided in three groups: ASD (*n* = 21), ASD + APS (*n* = 16), and HC (*n* = 20).

### Clinical Assessment

All ASD individuals underwent a cognitive evalution to assess IQ. Depending on the age and on each individual's ability to cooperate, the Leiter International Performance Scale—Revised (Leiter-R) (44), the Wechsler Intelligence Scale for Children—Fourth Edition (WISC-IV) (45), or the Wechsler Adult Intelligence Scale—Revised (WAIS-R) (46) was performed.

ASD diagnosis was based on the DSM-5 criteria (1). All participants underwent the Autism Diagnostic Observation Schedule—Second Edition (ADOS-2) test (47), performed by a licensed clinician. The ADOS-2, a semi-structured observational assessment of autistic symptoms, includes five modules based on expressive language level and age. The ADOS-2 algorithm is organized in social affect (SA), restricted and repetitive behaviors (RRB), and the total score. Modules 1, 2, and 3 provide the calibrated severity score (CSS), ranging from 1 to 10, indicating a measure of autism severity level. By contrast, even if a recent revision of the algorithm for module 4 is now available (48), this has not been applied yet to the Italian population. Thus, based on age, language, and adaptive abilities, all ASD subjects were administered module 3, being in line with the ADOS-2 manual (47) which allows clinicians and researchers to choose module 3 even for adolescents over 16 years of age in order to permit a comparison between CSS scores in the ASD sample (47, 49).

Finally, to evaluate the presence of a concurrent APS condition, the Structured Interview for Psychosis-Risk Syndromes (SIPS) was administered to all ASD individuals (50–52). In this context, the proposed “Attenuated Psychosis Syndrome (APS)” DSM-5 diagnosis refers to a condition characterized by the recent onset of sub-threshold psychotic symptoms associated to a higher risk of conversion to psychotic disorder within the next year (53–55).

According to a previous study (56), the presence of an APS condition was confirmed when a score of 3 or 4 of 5 was obtained at the SIPS positive symptoms scale (SIPS-P) (51, 55) (see Supplementary Figure 1 for SIPS-P score distributions in ASD and ASD + APS).

Moreover, the absence of a family history of schizophrenia was confirmed by the administration of a clinical interview.

Finally, the HC underwent a screening clinical evaluation of IQ [based on age, Raven Matrices colored (age < 11 years) or progressive (age > 11 years) (57)] and of behavioral problems (Child Behavior Checklist 6–18 years—CBCL 6–18 years) (58). Moreover, a clinical interview was performed by a child and adolescent psychiatrist in order to exclude the presence of autistic or psychotic symptoms in this population.

All the HC individuals presented normal cognitive function (IQ assessed with Raven Matrices above 25–50° centile for colored form and above 85 for the progressive form) and resulted negative for the presence of any behavioral problem or psychiatric condition.

### Neurophysiological Evaluation

All subjects (ASD, ASD + APS, and HC) underwent a 64-channel EEG recorded while listening to a passive auditory paradigm and watching a silent video. The MMN waveforms to frequency-deviant tones (*f-MMN*) and duration-deviant tones (*d-MMN*) were obtained for the Fz channel. The MMN peak latency and peak amplitude were measured. MMN is, in fact, described by *latency*, which is the timing of the negative peak in different waveforms, and *amplitude*, which represents the average response on the negative peak, reflecting attentive cortical processes (4).

The EEG was recorded at 2,048 Hz with an EBNeuro 72-channel EEG system using sintered Ag/Ag-Cl electrodes in an electrode cap with 61 standard scalp sites and two single Ag/AgCl disk electrodes (1 mm in diameter) over the left and the right mastoids, with the reference electrode placed between AFz and Fz and the ground electrode between CPz and Pz; one polygraphic channel was also recorded to monitor vertical and horizontal eye movements, with the active electrode placed 1 cm lateral to the outer canthus of the right eye and the reference electrode placed on the lower eyelid. The electrode impedances were maintained below 5 kΩ.

### Stimuli and Tasks

The auditory paradigm consisted of 2,000 standard tones (1,000 Hz, 50 ms), 200 frequency-deviant tones (1,200 Hz, 50-ms duration, 5-ms rise/fall), and 200 duration-deviant tones (1,000 Hz, 100 ms, 10-ms rise/fall), for a total of 2,400 tones. All tones were presented binaurally through Sennheiser HD25 SP II at 80 dB SPL with an interstimulus interval of 500 ms and in random order with the constraint that a deviant tone was preceded at minimum by three standard tones. During the auditory paradigm, the subjects were invited to watch a silent cartoon and to ignore the tones that they heard; at the end of the session, questions on the cartoon were asked to the participants in order to check the level of attention in watching the video.

### MMN Analysis

The EEG data were processed offline using EEGLAB in MATLAB environment. The continuous EEG recordings were digitally filtered 1 to 20 Hz, re-referenced to the algebraic average of the left and the right mastoids, and segmented from −100 to 400 ms relative to the stimulus onset. Epochs with artifacts exceeding ±100 μV were excluded. Average ERPs were computed from the artifact-free epochs and baseline-corrected with a prestimulus baseline of −100 to 0 ms.

The frequency and duration MMNs were measured at Fz channel as an average amplitude under within 100 to 200 ms (frequency) and 150 to 250 ms (duration) post-stimulus onset in the different waveform (deviant–standard), respectively.

### Statistical Analyses

Data were presented as means (SD) and frequencies (percentages). Differences in age and gender between HC, ASD, and ASD + APS were investigated, respectively, using one-way analysis of variance (ANOVA) and Pearson's chi-square test (χ^2^). Comparisons between ASD and ASD + APS in ADOS and SIPS measures were performed with Student's *t*-test.

Explorative data analysis with the Kolmogorov–Smirnov test showed that the MMN indices had a normal distribution (consistently, *p* > 0.20). ANOVA models were used to measure the difference between HC, ASD, and ASD + APS in d-MMN and f-MMN latencies and amplitudes. *Post-hoc* tests were performed with Bonferroni confidence interval adjustment for multiple comparisons to define which variables contributed to the major effects.

Explorative correlation analysis was performed in order to investigate the relation between MMN and clinical (ADOS and SIPS) indices. Statistical significance was set at *p*-values < 0.05.

## Results

### Demographic and Clinical Data

Age, sex, IQ, ADOS-2, and SIPS subscale scores are summarized in Table 1.

**Table 1:** Descriptive and univariate statistics of sociodemographic and clinical characteristics in healthy controls (HC) and autism spectrum disorder (ASD) and ASD + attenuated psychosis syndrome (ASD+APS) patients.

	**HC (*N* = 20)**	**ASD (*N* = 21)**	**ASD + APS (*N* = 16)**	**Statistics**
Gender (F/M)	8/12	4/17	4/12	*p* = 0.312
Age	14.20 (4.56)	14.28 (2.92)	14.00 (3.31)	*F*_2, 54_ = 0.028, *p* = 0.973
IQ		105.14 (17.77)	96.50 (19.49)	*t*_35_ = −1.406, *p* = 0.169
ADOS-2 SA	–	7.86 (2.52)	9.25 (3.13)	*t*_35_ = −1.501, *p* = 0.142
ADOS-2 RRB	–	1.67 (1.49)	2.50 (2.03)	*t*_35_ = −1.439, *p* = 0.159
ADOS-2 CSS	–	5.81 (1.54)	6.88 (2.09)	*t*_35_ = −1.787, *p* = 0.083
SIPS-P	–	2.38 (3.07)	6.44 (3.60)	*t*_35_ = −3.696, *p* = 0.0008
SIPS-N	–	3.29 (2.53)	3.81 (1.83)	*t*_35_ = −0.703, *p* = 0.487
SIPS-D	–	1.43 (1.50)	2.81 (0.91)	*t*_35_ = −3.252, *p* = 0.003
SIPS-G	–	1.38 (1.12)	2.19 (1.05)	*t*_35_ = −2.235, *p* = 0.032
SIPS total	–	8.48 (5.02)	15.25 (3.53)	*t*_35_ = −4.597, *p* < 0.0001

*Values are frequencies and means (and SDs)*.

*IQ, intelligence quotient; ADOS-2: Autism Diagnostic Observation Schedule—Second Edition; ADOS-2 SA, social affect domain; ADOS-2 RRB, restricted and repetitive behaviors; ADOS-2 CSS, calibrated severity score; SIPS: Structured Interview for Psychosis-Risk Syndromes; SIPS-P, positive symptoms domain; SIPS-N, negative symptoms domain; SIPS-D, disorganization symptoms domain; SIPS-G, general symptoms domain*.

No significant differences emerged in terms of age [ASD vs. HC: 14.16 (3.05) vs. 14.20 (4.56); *t*_55_ = −0.040, *p* = 0.969] and sex ($\chi_{1}^{2}$ = 2.172, *p* = 0.141). Moreover, within the ASD sample, no differences in terms of IQ came out (see Supplementary Figure 2).

At the clinical level, the ASD + APS group exhibits a higher level of psychotic symptoms as assessed by the SIPS interview compared to the ASD (SIPS total, *p* < 0.0001). Specifically, compared to the ASD, in the ASD + APS group, we observe a higher value of positive (SIPS-P, *p* = 0.001), disorganized (SIPS-D, *p* = 0.002), and general psychotic symptoms (SIPS-G, *p* = 0.031).

However, no relevant differences emerged between the two groups in terms of negative symptoms (SIPS-N, *p* = 0.468) and autistic features measured by the ADOS-2 test (ADOS-2 CSS, *p* = 0.098).

### MMN Component Characteristics

**Figure 1:**
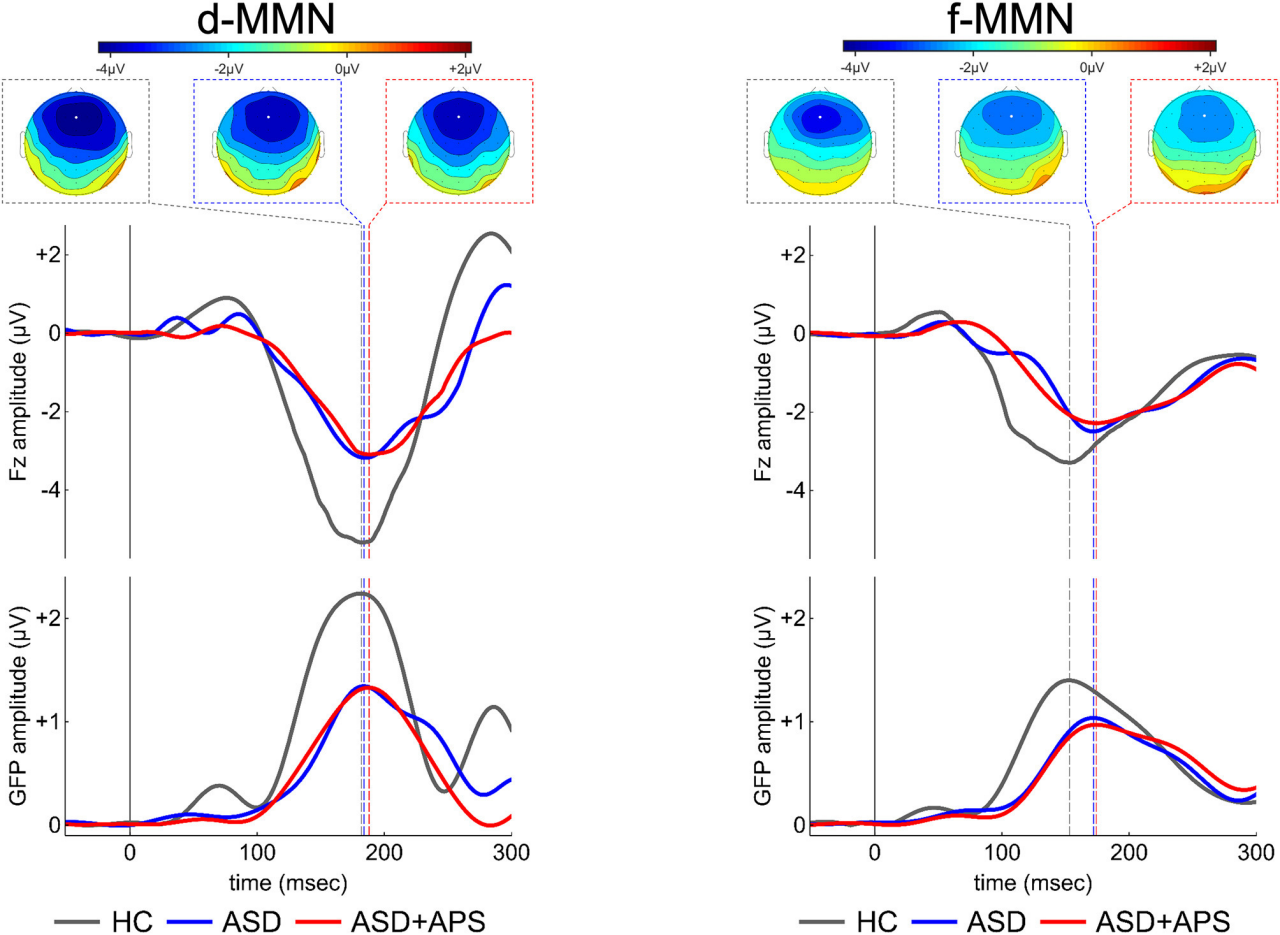
Electrical scalp map topographies (for each group, at the MMN grandaverage peak; upper panels portion), grandaverage waveforms at Fz channel **(middle panels portion)** and Global Field Power (GFP, a global measure of electrical field strength; **middle panels portion**) in the three groups (ASD, ASD+APS, and HC) for each condition, d-MMN **(left panel)** and f-MMN **(right panel)**. As expected, MMN has a fronto-central distribution over the scalp midline, with strongest electrical field responses around Fz channel (indicated as a white circle in the scalp map images). ASD: Autism Spectrum disorder; ASD+APS: Autism Spectrum Disorder + Attenuated Psychosis Syndrome; HC: Healthy Controls.

Compared to HC, the whole ASD group showed significantly less negative values of d-MMN amplitude [−4.40 (2.02) vs. −6.72 (1.70); *t*_55_ = 4.355, *p* < 0.0001] as well as longer f-MMN latency [173.76 (29.50) vs. 153.80 (13.13); *t*_55_ = 2.867, *p* = 0.006] and less negative values of f-MMN amplitude [−3.43 (1.88) vs. −4.80 (1.33); *t*_55_ = 2.900, *p* = 0.005] (Table 2; Figure 1).

**Table 2:** Descriptive and univariate statistics of dMMN and fMMN latency and amplitude in healthy controls (HC) and autism spectrum disorder (ASD) and ASD + attenuated psychosis syndrome (ASD+APS) patients.

	**HC (*N* = 20)**	**ASD (*N* = 21)**	**ASD + APS (*N* = 16)**	**ANOVAs**			
				**Main effect**	**Bonferroni** ***post-hoc***		
					**HC vs. ASD**	**HC vs. ASD + APS**	**ASD vs. ASD + APS**
**d-MMN**							
Latency (ms)	182.70 (15.00)	184.62 (21.70)	189.25 (30.43)	*F*_2, 54_ = 0.386, *p* = 0.682	*p* = 1.000	*p* = 1.000	*p* = 1.000
Amplitude (μV)	−6.72 (1.70)	−4.53 (2.00)	−4.22 (2.10)	*F*_2, 54_ = 9.471, *p* = 0.0003	*p* = 0.002	*p* = 0.0009	*p* = 1.000
**f-MMN**							
Latency (ms)	153.80 (13.13)	172.62 (26.13)	175.25 (34.27)	*F*_2, 54_ = 4.090, *p* = 0.022	*p* = 0.062	*p* = 0.043	*p* = 1.000
Amplitude (μV)	−4.80 (1.33)	−3.57 (1.83)	−3.24 (1.98)	*F*_2, 54_ = 4.317, *p* = 0.018	*p* = 0.076	*p* = 0.027	*p* = 1.000

d-MMN, duration-deviant tones mismatch negativity; f-MMN, frequency-deviant tones mismatch negativity.

No difference between ASD and HC in d-MMN latency [186.62 (25.55) vs. 182.70 (15.00); *t*_55_ = 0.629, *p* = 0.532] came out.

The ANOVA models showed that the three groups were different in d-MMN amplitudes and f-MMN latencies and amplitudes. Specifically, the *post-hoc* analysis showed that ASD and ASD + APS had higher values in latencies and lower values in amplitudes with respect to HC. However, in terms of MMN indexes, no significant differences came out between the ASD and the ASD + APS groups (Table 2). Moreover, including the IQ as a covariate, no significant differences emerged between the two groups (see Supplementary Tables 1, 2).

### Correlation Between MMN Component Characteristics and Clinical Data

Correlation coefficients, with Bonferroni-adjusted *P*-values (*p*-value threshold / number of comparisons = 0.05/36 = 0.0013889 = 0.0014), were computed between d-MMN and f-MMN amplitude and latency indices and ADOS-2 and SIPS subscale scores separately in the ASD and the ASD + APS groups (Table 3).

**Table 3:** Results of correlation analysis between mismatch negativity indices and clinical phenotype in autism spectrum disorder (ASD) and ASD + attenuated psychosis syndrome (ASD+APS) groups are reported.

	**SA**	**ADOS-2 RRB**	**ADOS-2 CSS**	**SIPS-P**	**SIPS-N**	**SIPS-D**	**SIPS-G**	**SIPS-tot**
***ASD***								
*dMMN_Fz_latency*	*r* = −0.197	*r* = −0.400	*r* = *−0.457*	*r* = 0.155	*r* = 0.150	*r* = 0.028	*r* = 0.400	*r* = 0.268
	*p* = 0.392	*p* = 0.072	*p* = *0.037*	*p* = 0.502	*p* = 0.518	*p* = 0.903	*p* = 0.072	*p* = 0.240
*dMMN Fz amplitude*	*r* = −0.061	*r* = 0.132	*r* = 0.051	*r* = −0.148	*r* = −0.358	*r* = −0.219	*r* = −0.294	*r* = −0.403
	*p* = 0.792	*p* = 0.568	*p* = 0.826	*p* = 0.522	*p* = 0.111	*p* = 0.341	*p* = 0.195	*p* = 0.070
*fMMN Fz latency*	*r* = −0.101	*r* = 0.478	*r* = 0.185	*r* = 0.152	*r* = 0.015	*r* = 0.131	*r* = 0.134	*r* = 0.169
	*p* = 0.665	*p* = 0.0*28*	*p* = 0.422	*p* = 0.511	*p* = 0.950	*p* = 0.573	*p* = 0.563	*p* = 0.463
*fMMN Fz amplitude*	*r* = 0.316	*r* = 0.043	*r* = 0.371	*r* = −0.227	*r* = −0.071	*r* = −0.151	*r* = −0.148	*r* = −0.254
	*p* = 0.163	*p* = 0.852	*p* = 0.098	*p* = 0.322	*p* = 0.759	*p* = 0.512	*p* = 0.521	*p* = 0.268
***ASD+APS***								
*dMMN Fz latency*	*r* = *−0.575*	*r* = −0.426	*r* = *−0.650*	*r* = 0.076	*r* = 0.094	*r* = −0.010	*r* = −0.029	*r* = 0.115
	*p* = 0.0*20*	*p* = 0.100	*p* = 0.0*06*	*p* = 0.780	*p* = 0.729	*p* = 0.970	*p* = 0.916	*p* = 0.672
*dMMN Fz amplitude*	*r* = −0.420	*r* = −0.108	*r* = −0.232	*r* = −0.193	*r* = −0.059	*r* = 0.139	*r* = 0.400	*r* = −0.073
	*p* = 0.106	*p* = 0.690	*p* = 0.387	*p* = 0.473	*p* = 0.830	*p* = 0.611	*p* = 0.124	*p* = 0.788
*fMMN Fz latency*	*r* = *−0.616*	*r* = *−0.535*	*r* = *−0.811*	*r* = −0.090	*r* = 0.024	*r* = −0.122	*r* = −0.282	*r* = −0.195
	*p* = 0.0*11*	*p* = 0.0*33*	*p* = 0.0*00*	*p* = 0.740	*p* = 0.929	*p* = 0.652	*p* = 0.290	*p* = 0.470
*fMMN Fz amplitude*	*r* = −0.309	*r* = 0.097	*r* = −0.125	*r* = 0.095	*r* = 0.196	*r* = 0.108	*r* = 0.300	*r* = 0.315
	*p* = 0.244	*p* = 0.722	*p* = 0.645	*p* = 0.727	*p* = 0.467	*p* = 0.690	*p* = 0.259	*p* = 0.235

*Significant results are shown in italics*.

*ADOS-2: Autism Diagnostic Observation ScheduleSecond Edition; ADOS-2 SA, social affect domain; ADOS-2 RRB, restricted and repetitive behaviors; ADOS-2 CSS, calibrated severity score; SIPS-tot, SIPS total score; SIPS: Structured Interview for Psychosis-Risk Syndromes; SIPS-P, positive symptoms domain; SIPS-N, negative symptoms domain; SIPS-D, disorganization symptoms domain; SIPS-G, general symptoms domain; p, Bonferroni adjusted p-values (statistical significance at p-values < 0.05)*.

Specifically, in the ASD group (Table 3), we found a negative correlation between d-MMN latency and ADOS-2 CSS score (*r* = −0.457; *p* = 0.037) and a positive correlation between f-MMN and ADOS-2 RRB (*r* = 0.478; *p* = 0.028).

**Figure 2:**
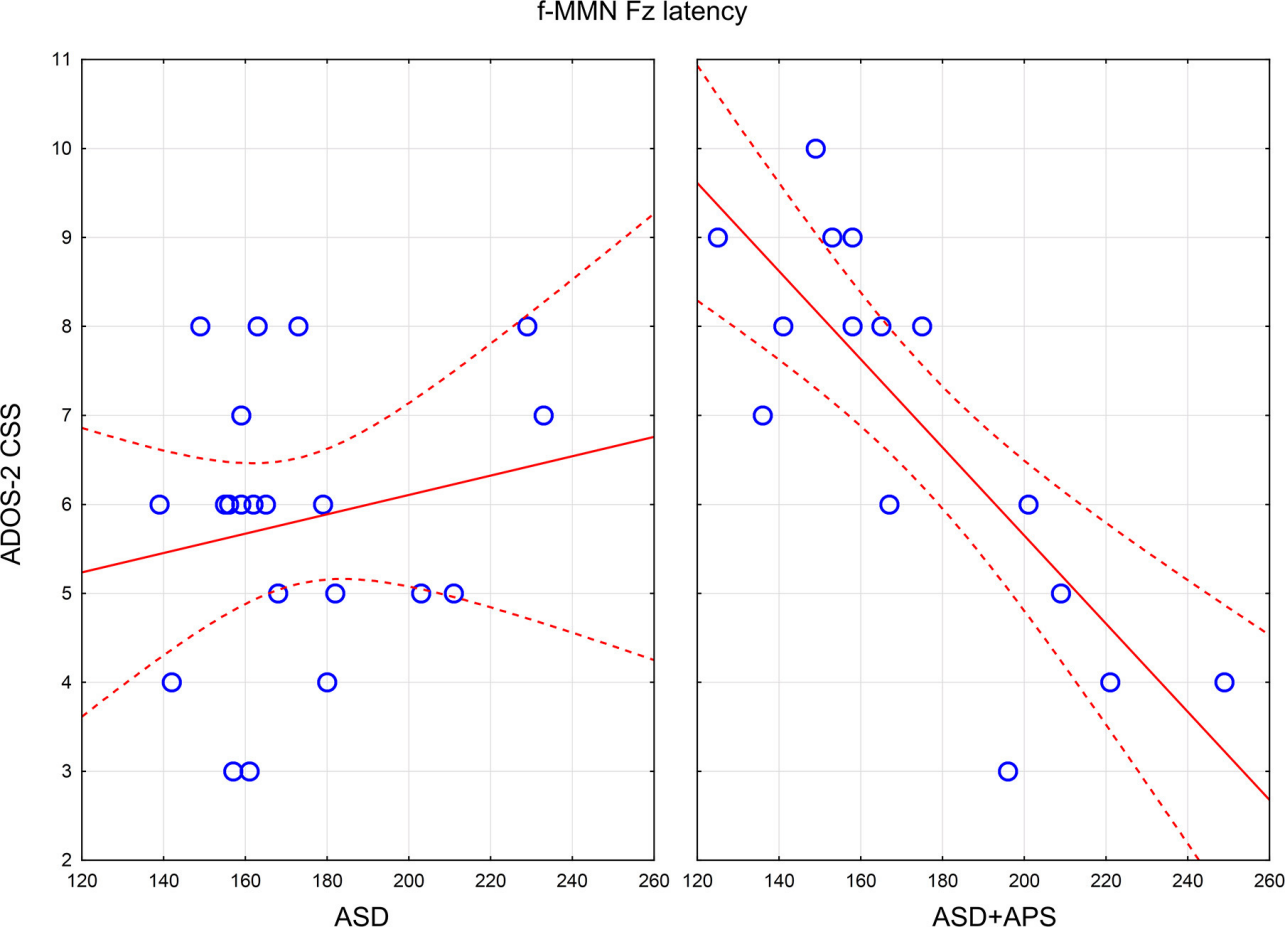
Scatterplots of relations between f-MMN latency and ADOS-2 CSS in ASD **(left panel)** and ASD+APS **(right panel)**. In the panels, the continuous lines are the linear regression lines, the dashed lines indicate the 95% Confidence Intervals for mean, and the empty circles represent the value for each autistic subject. ADOS-2 CSS: Autism Diagnostic Observation ScheduleSecond Edition, calibrated severity score; ASD: Autism Spectrum disorder; ASD+APS: Autism Spectrum Disorder + Attenuated Psychosis Syndrome; f-MMN: frequency-deviant tones mismatch negativity.

In the ASD + APS group (Table 3), applying Bonferroni correction (*p*-value threshold / number of comparisons = 0.05/36 = 0.0013889 = 0.0014), a strong negative correlation emerged between f-MMN latency and ADOS-2 CSS (*r* = −0.811; *p* < 0.0001), RRB (*r* = −0.535; *p* = 0.033), and SA (*r* = −0.616; *p* = 0.011) and between d-MMN latency and ADOS-2 CSS (*r* = −0.650; *p* = 0.006) and ADOS-2 SA (*r* = −0.575; *p* = 0.020), demonstrating that a higher level of autistic symptoms was linked to reduced MMN latencies (Figure 2).

No significant correlation emerged between MMN amplitude and latency parameters to SIPS scores in both groups (ASD and ASD + APS).

## Discussion

MMN has been well-established as a good reliable marker for pre-attentional mechanism. However, during the past years, MMN has been separately studied in the autistic (16, 17) and in the at-risk psychotic population (20, 21, 59), and findings of the concurrent presence of both ASD and APS have not been reported so far. Thus, in the present study, we aimed to investigate differences in MMN features concerning both frequency and duration deviants in a pediatric sample of ASD participants with or without co-occurrent APS compared to a healthy control group.

Being in line with a recent meta-analysis on the topic by Chen et al. (17), showing a reduced amplitude in response to non-speech sound deviants in the ASD population, our results support the evidence that young ASD individuals present a different pattern of pre-attentional processes measured as MMN amplitude indexes compared to HC. By contrast, our data demonstrate a prolonged MMN latency in response to tone–frequency deviants in the whole ASD group, as reported in AS individuals. As is known, previous research also suggests a key role of the brain temporal regions in either processing language and social cognition abilities (60–62) or auditory memory processes in ASD individuals (63). Taking these results that refer to available studies together, our findings yield the knowledge that ASD individuals present a significant impairment in pre-attentive temporal auditory processing (64), showing a slower response in the sound duration identification task (65).

Additionally, we have evaluated the MMN paradigm not only in ASD participants but also in individuals with co-occurrent APS in order to investigate whether the presence of concurrent attenuated psychotic symptoms could influence the pre-attentive pattern in this “at-risk population.” Our results show that, when an APS condition is associated to ASD, no significant differences emerged in terms of MMN indexes, leading to the hypothesis that, in autistic individuals, the presence of a concurrent APS condition does not significantly impair the pre-attentive temporal auditory process measured at MMN.

Furthermore, to better investigate the differences between the two groups (ASD and ASD + APS) and the impact of the clinical phenotype, we analyzed the correlations between MMN indexes to both autistic and psychotic symptoms. Our results demonstrate a significant correlation between electrophysiological indexes, referred to MMN latencies, and autistic symptoms level. Specifically, in the ASD group, we found a negative correlation between d-MMN latency and ADOS-2 CSS score, with reduced latency in response to duration deviants linked to a higher autistic symptom level. In the ASD + APS group, we noticed an even stronger negative correlation between ADOS-2 CSS score and both d-MMN and f-MMN latency indexes, with reduced MMN latency associated with a higher level of autistic symptoms. No significant correlations to attenuated psychotic symptoms measured by the SIPS interview came out. Interestingly, it is known that a shorter MMN latency, generated by both frequency and duration tone deviants, is described in individuals affected with schizophrenia (66–68) and in those considered at risk for psychosis (59), without a significant correlation to the severity of the psychotic symptoms (68). Thus, from a neurobiological perspective, if the earlier peak of MMN could reflect temporal responsiveness in the auditory cortex, both in ASD and ASD + APS populations (69), from a clinical point of view, the presence of a strong negative correlation between the latency indexes in both deviants (d-MMN and f-MMN) to the severity of autistic symptoms in the ASD + APS group could describe a greater clinical impairment in this population, especially referring to autistic symptoms. Moreover, given that other studies (21, 70–72) reported that different MMN latency features, in both d-MMN and f-MMN, could be a possible biomarker in different stages of psychotic symptoms, it is crucial to further investigate whether the duration of the illness could impact on the correlation between MMN features and clinical phenotype. Thus, the next step of this research study will be the differentiation, in terms of MMN latency parameters, between ASD + APS subjects who would convert to psychosis and those who would not.

Although in ASD individuals it is still challenging to translate neurophysiological indexes into clinically prognostic features, understanding the role of MMN as a possible biomarker of psychotic symptom progression could help clinicians to better describe ASD individuals at risk for psychosis. In this context, our findings start to provide a more informative characterization of the ASD sub-phenotype by a dimensional approach and a better evaluation of illness progression within individuals considered “at-risk” for developing psychotic disorders. This knowledge might consequently lead to an optimal management of the therapeutic intervention in terms of choice, timing, and duration.

Thus, despite some strengths such as the clinically well-described ASD sample and the inclusion of young individuals, our study presents some limitations that should be taken into account when interpreting our data. Firstly, the sample size. Even if our study is in line with the majority of available studies aimed to assess MMN in ASD individuals (17), the sample size is relatively small if compared to other clinical studies [see Foss-Feig et al. (73)]. Secondly, the age range (9–18 years). Indeed even if including young individuals in the sample is a strength of the study, allowing to assess the presence of psychosis risk at a very early prodromal phase (74), the exclusion of individuals aged over 18 years old does not permit to explore the possible onset of psychotic symptoms in the ASD group at that point. Moreover, the evaluation of psychotic symptoms in young autistic individuals with cognitive borderline functioning could be challenging, even for trained clinicians. Besides, the lack of information about the age onset of psychotic symptoms in ASD individuals before our assessment is a limitation. Furthermore, the lack of an APS control group without ASD, in order to better evaluate the significance of our results in the ASD + APS group, is another limiting factor. Finally, it is important to highlight a possible recruitment bias in the fact that we included all participants with well-established compliance. This strongly highlights the need for further investigations, especially through longitudinal studies, in order to better evaluate the possible relevance and the prognostic meaning of the MMN preattentive patterns deficit in ASD individuals at risk for psychosis.

## Data Availability Statement

The data that support the finding of this study are available on request from the corresponding authors, GDL and LM.

## Ethics Statement

The studies involving human participants were reviewed and approved by Ethical Committee of our University Hospital, Fondazione Policlinico Tor Vergata, Rome (Register number 126/18). Written informed consent to participate in this study was provided by the participants' legal guardian/next of kin.

## Author Contributions

LM, GDL, and MR conceived and designed the present study. AR and MS evaluated autism spectrum disorder and assessed the symptom severity. MR evaluated the psychotic symptoms. GDL performed the electrophysiological recordings and analyzed the data. GDL, AR, and MS wrote the manuscript, and LM and PC substantially revised the manuscript. All the authors contributed to the writing of the manuscript.

## Conflict of Interest

The authors declare that the research was conducted in the absence of any commercial or financial relationships that could be construed as a potential conflict of interest.
