# Supplementary figures and images for: Auditory Mismatch Negativity in Youth Affected by Autism Spectrum Disorder With and Without Attenuated Psychosis Syndrome
Source: Front Psychiatry. 2020 Nov 24;11:555340. doi: 10.3389/fpsyt.2020.555340 (PMC7732489; doi:10.3389/fpsyt.2020.555340)

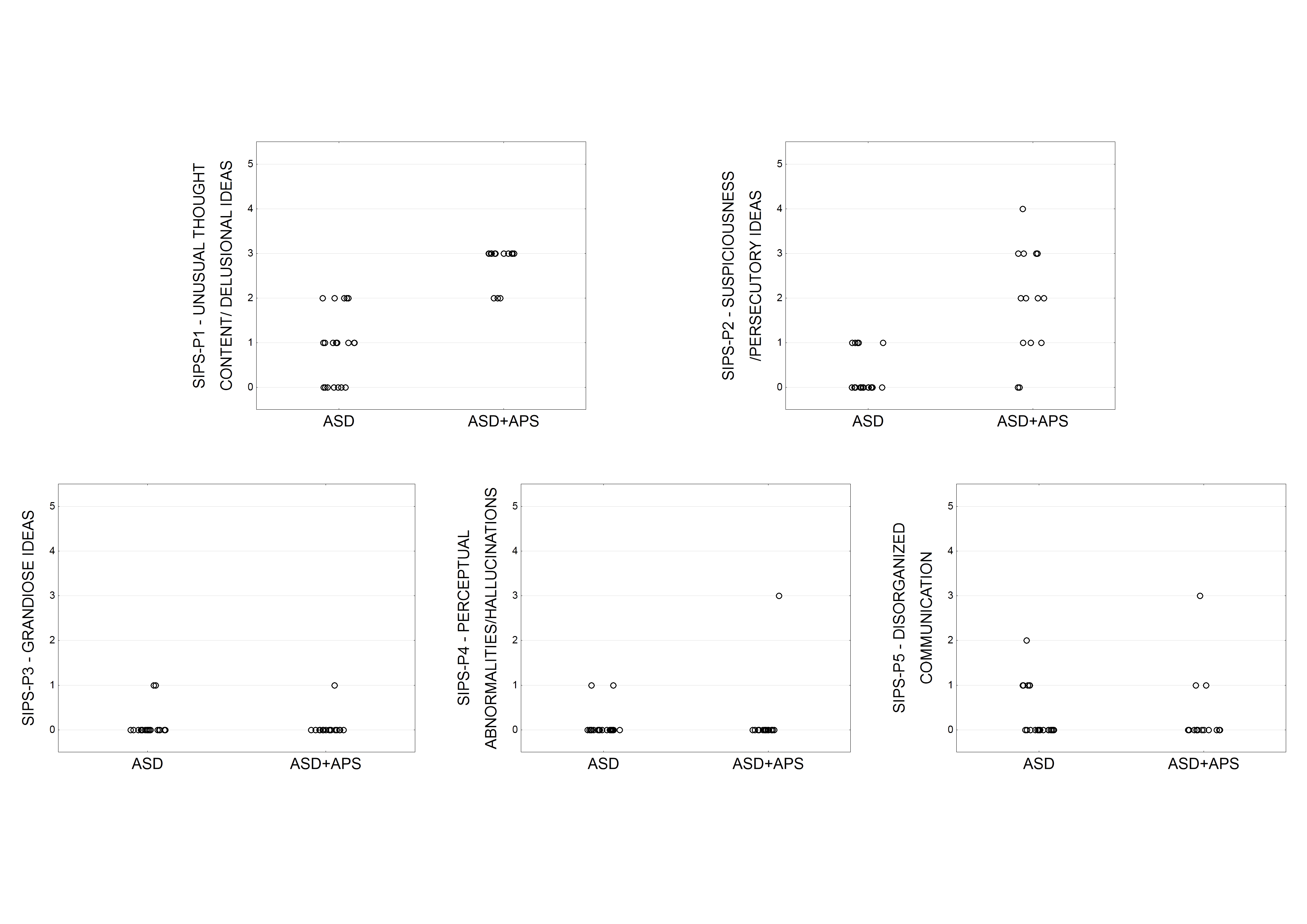

Supplement: Supplementary file 1 [file Image_1.tiff]

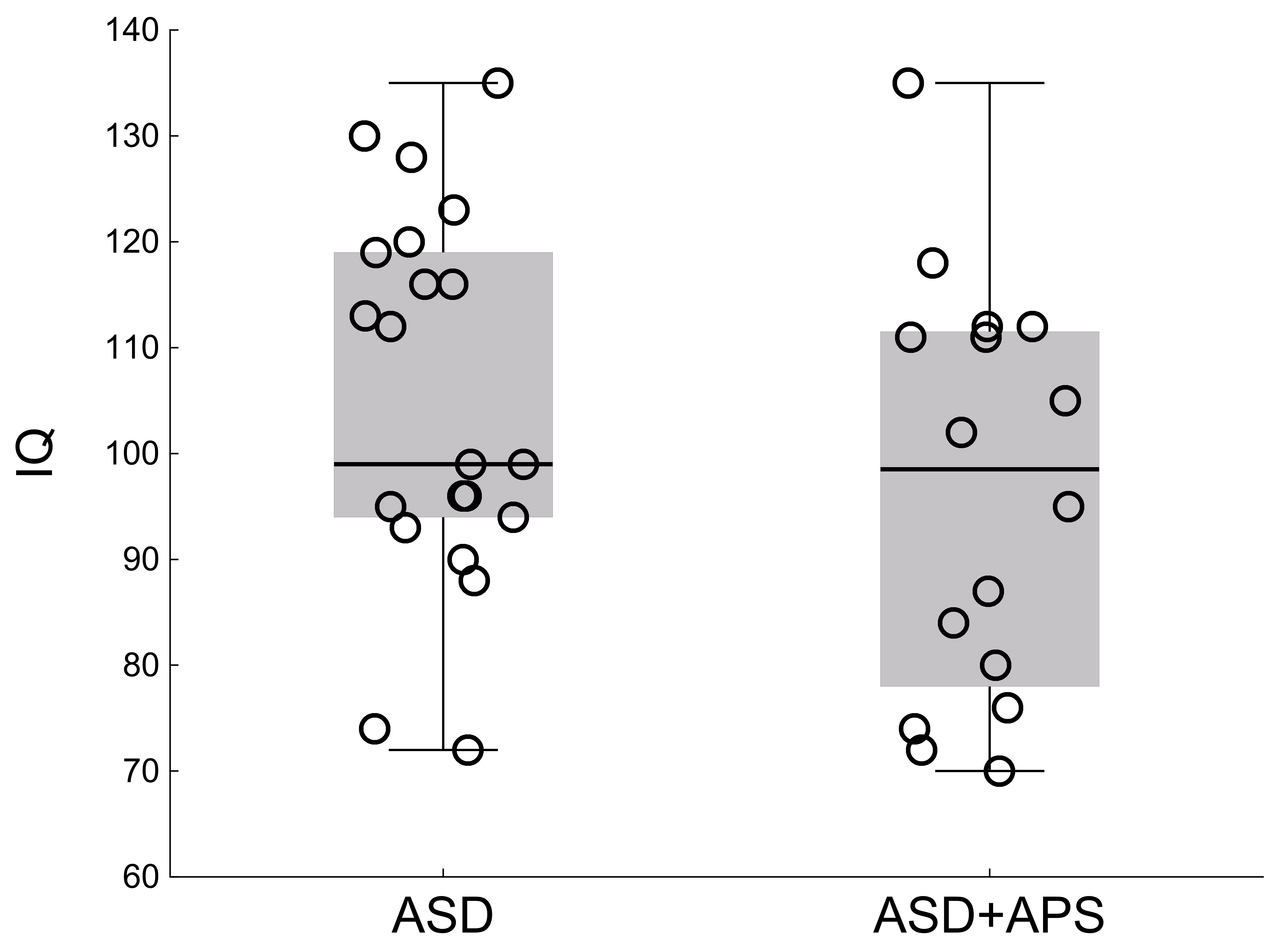

Supplement: Supplementary file 2 [file Image_2.tif]
