# Supplementary material for: Auditory Mismatch Negativity in Youth Affected by Autism Spectrum Disorder With and Without Attenuated Psychosis Syndrome
Source: Front Psychiatry. 2020 Nov 24;11:555340. doi: 10.3389/fpsyt.2020.555340 (PMC7732489; doi:10.3389/fpsyt.2020.555340)
Supplement: Supplementary file 3 [file Data_Sheet_1.PDF]

Methods and main results

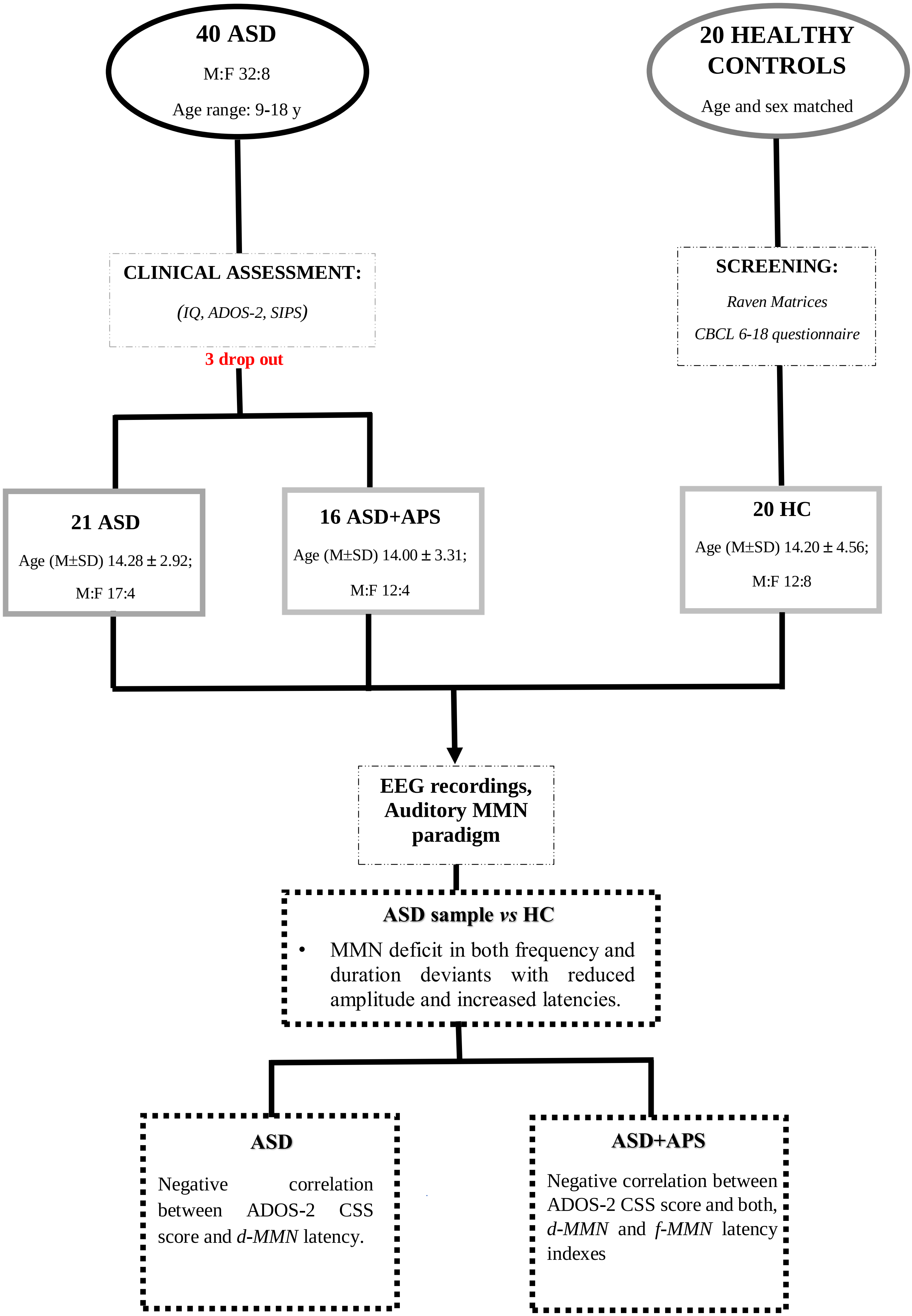

Legend:

ASD: Autism Spectrum Disorder
APS: Attenuated Psychosis Syndrome
HC: Healthy Controls
MMN: Mismatch Negativity
fMMN: Mismatch Negativity frequency deviant
dMMN: Mismatch Negativity duration deviant
IQ: Intelligence Quotient
ADOS-2: Autism Diagnostic Observation Schedule – Second Edition ADOS-2
SIPS: Structured Interview for Prodromal Syndromes
CBCL 6-18 years: *Child Behaviour Checklist 6-18 years*
