# Supplementary material for: Auditory Mismatch Negativity in Youth Affected by Autism Spectrum Disorder With and Without Attenuated Psychosis Syndrome
Source: Front Psychiatry. 2020 Nov 24;11:555340. doi: 10.3389/fpsyt.2020.555340 (PMC7732489; doi:10.3389/fpsyt.2020.555340)
Supplement: Supplementary file 4 [file Data_Sheet_2.docx]

**Supplementary Table 1**. Results of ANOVA and ANCOVA models with MMN indices as dependent variable, ASD (n=21) vs. ASD+APS (n=16) as between-subject factor, and IQ total score as covariate.

|  |  | **ANOVA** | | |  | **ANCOVA** | | |
| --- | --- | --- | --- | --- | --- | --- | --- | --- |
|  |  | F | df | p |  | F | df | p |
| **d-MMN** |  |  |  |  |  |  |  |  |
| latency (ms) |  | 0.069 | 1,35 | 0.794 |  | 0.565 | 1,34 | 0.457 |
| amplitude (µV) |  | 0.042 | 1,35 | 0.838 |  | 0.214 | 1,34 | 0.646 |
| **f-MMN** |  |  |  |  |  |  |  |  |
| latency (ms) |  | 0.003 | 1,35 | 0.954 |  | 0.085 | 1,34 | 0.773 |
| amplitude (µV) |  | 0.031 | 1,35 | 0.862 |  | 0.014 | 1,34 | 0.908 |

Legend: ANOVA: analysis of variance; ANCOVA: analysis of covariance; ASD: Autism Spectrum disorder; ASD+APS: Autism Spectrum Disorder + Attenuated Psychosis Syndrome; d-MMN: duration-deviant tones mismatch negativity; f-MMN: frequency-deviant tones mismatch negativity; IQ: Intelligence Quotient.

**Supplementary Table 2**. Correlations between total IQ and dMMN and fMMN latency and amplitude in ASD and ASD+APS.

|  | IQ | | |
| --- | --- | --- | --- |
|  | ASD  (N=21) | ASD+APS  (N=16) | |
|  |  |  |  |
|  |  |  |  |
| **d-MMN** |  |  |  |
| latency (ms) | r=.350, p=.120 | r=.328, p=.216 |  |
| amplitude (µV) | r=.045, p=.848 | r=.371, p=.157 |  |
| **f-MMN** |  |  |  |
| latency (ms) | r=.122, p=.598 | r=.220, p=.413 |  |
| amplitude (µV) | r=-.036, p=.879 | r=-.043, p=.876 |  |

Legend: ASD: Autism Spectrum disorder; ASD+APS: Autism Spectrum Disorder + Attenuated Psychosis Syndrome; d-MMN: duration-deviant tones mismatch negativity; f-MMN: frequency-deviant tones mismatch negativity; IQ: Intelligence Quotient.
